# Identification of uterine pacemaker regions at the myometrial–placental interface in the rat

**DOI:** 10.1113/JP275688

**Published:** 2018-05-30

**Authors:** E. Josiah Lutton, Wim J. E. P. Lammers, Sean James, Hugo A. van den Berg, Andrew M. Blanks

**Affiliations:** ^1^ Cell and Developmental Biology Division of Biomedical Sciences Warwick Medical School University of Warwick Coventry CV4 7AL UK; ^2^ Bioengineering Institute Auckland University Auckland New Zealand; ^3^ Department of Physiology College of Medicine and Health Sciences UAE University Al Ain United Arab Emirates; ^4^ Department of Pathology University Hospitals Coventry and Warwickshire (UHCW) NHS Trust Coventry CV2 2DX UK; ^5^ Warwick Mathematics Institute University of Warwick Coventry CV4 7AL UK

**Keywords:** myometrium, uterus, pregnancy, labour, pacemaker, parturition

## Abstract

**Key Points:**

- Coordinated contraction of the uterine smooth muscle is essential to parturition.
- Histologically and physiologically defined pacemaker structures have not been identified in uterine smooth muscle.
- Here we report combined electrophysiological and histological evidence of zones associated with pacemaker activity in the rat myometrium.
- Our method relies crucially on the integration of histological and electrophysiological data in an* in silico* three‐dimensional reconstruction of the rat myometrium at 10 μm resolution.
- We find that myometrial/placental pacemaking zones are closely related with placental sites and the area of disruptive myometrial remodelling surrounding such sites.
- If analogues of the myometrial/placental pacemaking zone are present in the human, defining their histology and physiology will be important steps towards treatment of pre‐term birth, pre‐eclampsia, and postpartum haemorrhage.

**Abstract:**

Coordinated uterine contractions are essential for delivering viable offspring in mammals. In contrast to other visceral smooth muscles, it is not known where excitation within the uterus is initiated, and no defined pacemaking region has hitherto been identified. Using multi‐electrode array recordings and high‐resolution computational reconstruction of the three‐dimensional micro‐structure of late pregnant rat uterus, we demonstrate that electrical potentials are initiated in distinct structures within the placental bed of individual implantation sites. These previously unidentified structures represent modified smooth muscle bundles that are derived from bridges between the longitudinal and circular layers. Coordinated implantation and encapsulation by invading trophoblast give rise to isolated placental/myometrial interface bundles that directly connect to the overlying longitudinal smooth muscle layer. Taken together, these observations imply that the anatomical structure of the uterus, combined with site‐specific implantation, gives rise to emergent patterns of electrical activity that drive effective contractility during parturition.

## Key Points

- Coordinated contraction of the uterine smooth muscle is essential to parturition.
- Histologically and physiologically defined pacemaker structures have not been identified in uterine smooth muscle.
- Here we report combined electrophysiological and histological evidence of zones associated with pacemaker activity in the rat myometrium.
- Our method relies crucially on the integration of histological and electrophysiological data in an* in silico* three‐dimensional reconstruction of the rat myometrium at 10 μm resolution.
- We find that myometrial/placental pacemaking zones are closely related with placental sites and the area of disruptive myometrial remodelling surrounding such sites.
- If analogues of the myometrial/placental pacemaking zone are present in the human, defining their histology and physiology will be important steps towards treatment of pre‐term birth, pre‐eclampsia, and postpartum haemorrhage.

## Introduction

Myometrial smooth muscle is capable of generating phasic contractions in the absence of stimuli from the central nervous system or circulating hormones (Garfield & Maner, 2007). As in all visceral muscles, contractions require the generation and propagation of electrical signals at the plasma membrane of cells, which are arranged in an electrotonically connected syncytium (Garfield *et al*. 1988). Tissues such as the heart and the stomach orchestrate electrical activity via dedicated anatomical structures that generate pacemaking potentials (Lammers *et al*. 2009). In contrast, the small intestine and bladder generate excitatory potentials at distinct but anatomically variable sites throughout the tissue (Lammers & Stephen, 2008; Hammad *et al*. 2014). To date, no pacemaker regions with specific anatomical features have been described in the mammalian uterus, which is perhaps surprising given that coordinated forceful contractions are essential to parturition. Multi‐electrode recordings of rat uteri demonstrated that potentials tend to initiate either at placental sites on the mesometrial border, or near the ovarian end (Lammers *et al*. 2015).

To shed light on the processes underlying activation in the rat myometrium, we established a three‐step procedure that combines isochronal analysis of multi‐electrode recordings with scans of histological slides of the same tissue specimens and automated image processing based on detection of cell nuclei, followed by 3D tissue reconstruction. This analysis enabled the correlation of the kinematics of reconstructed wave fronts of electrical activity propagating along the myometrium with a detailed reconstruction of tissue micro‐architecture, at an unprecedented resolution level of 10 μm for an entire organ measuring approximately 5 cm in length (Lutton *et al*. 2017). Using this strategy, we identified a defined histological structure in which electrical potentials are triggered by the integration of fetal and maternal stimuli, the ‘myometrial–placental pacemaker zone’ (MPPZ).

## Methods

### Ethical approval

Animal experiments were carried out at the University of Al‐Ain (institutional ethical approval: AE/03/30) in accordance with the local Institutional Animal Care and Use Committee regulations, the Animals (Scientific Procedures) Act and *The Journal of Physiology*’s guidelines on animal ethics. Virgin Wistar rats (*n *= 3) were time‐mated, and pregnancy dated as day 0 of gestation if the sperm cells were observed in the vaginal lavage the next morning. Rats had access to food and water *ad libitum* and were housed in a climate‐controlled room under a 12:12 h light–dark cycle. Rats were euthanized (D19‐20) by graded CO_2_ inhalation, a Schedule 1 procedure, and the uterine horns were rapidly excised via a midline incision of the abdomen.

### Electrophysiology

The uterine horns were opened longitudinally along the anti‐mesometrial border and pinned with the serosal side facing upwards to a final dimension of approximately 20 mm by 50 mm. The tissue was then perfused by placing in modified Tyrode solution (mm: 130 NaCl, 4.5 KCl, 2.2 CaCl_2_, 0.6 MgCl_2_, 24.2 NaHCO_3_, 1.2 NaH_2_PO_4_, and 11 glucose, saturated with carbogen (95% O_2_–5% CO_2_) pH 7.35, 37 °C) at a rate of 100 ml min^−1^. The electrophysiological experiments were performed as described in detail by Lammers *et al*. (2015). Electrical recordings were made using a custom rectangular 240‐electrode array (24 by 10; 2 mm interelectrode distance), which covered the entire preparation. The electrodes consisted of Teflon‐coated silver wires (0.3 mm diameter, Cooner Wire, Chatsworth, CA, USA). Unipolar electrograms were recorded from each individual electrode, with a silver plate located in the tissue bath serving as the common reference electrode. All electrodes were connected through shielded wires to 240 AC amplifiers where the signals were amplified (4000 times), filtered (bandwidth 2–400 Hz), digitized (8 bits, 1 kHz sampling rate) and stored on a PC. Recordings were performed for 30 consecutive minutes. After the experiments, signals were digitally filtered (using a 20‐point moving average) and displayed on screen. The beginning of every burst was located in time and the location of the first electrical signal in every burst noted.

### Analysis of multi‐electrode array recordings

**Figure 1:**
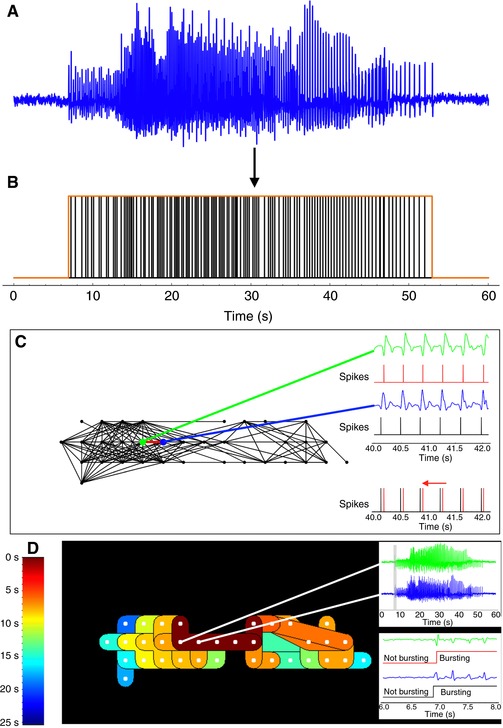
Processing pipeline for EMG data analysis Data from a 240 microelectrode array were acquired from the serosal myometrial surface over a 30 min period. *A*, an example of raw burst data acquired from an electrode in a time series. *B*, burst data were first processed by a combination of filtering and thresholding to yield an electrogram defining a burst duration (red box) and a set of action potential spike times (black spikes) to be used in a subsequent burst correlation algorithm. *C*, each defined burst was correlated across the electrode array to match electrodes that had detected the same burst to create propagation maps. Processed spike times were automatically identified for each electrogram, and compared over 2 s intervals. Comparisons were performed by translating the spikes backwards in time to identify a time displacement that aligned the spikes to within 20 ms. This process eliminated the possibility of observing independent burst events at different electrodes and misinterpreting the time difference of the events as propagation. Electrodes (dots) in the map are depicted as matched by connected lines, whereby connected electrodes detected the same burst during propagation. *D*, isochrone maps were generated from the propagation map in *C*. Burst initiation times for each electrode in a matched series were used to apply a pseudo‐coloured scale of 200 ms time bins. Thus, all electrodes that are depicted as the same colour recorded a matched burst of electrical activity *within* the same 200 ms time frame, though the time resolution of the recording and algorithm were still sufficient to infer propagation. In all subsequent isochrone maps, the electrode that first recorded an observed burst is marked by a white star.

Multi‐electrode array recordings were processed in order to determine initiation and propagation of excitation waves: bursting behaviour was characterized at each electrode by analysing the electrical activity recorded by the given electrode, and this behaviour was subsequently cross‐correlated across the electrode array in order to generate isochrone maps charting the propagation of the activity over the entire organ (Fig. 1).

Raw signals were processed by applying a Gaussian filter of radius 20 ms (Burden & Faires, 2005). Peaks and troughs in the smoothed signal were identified by applying seven‐point first‐ and second‐order numerical derivative filters (Burden & Faires, 2005). The peak‐to‐peak difference between each trough and subsequent peak was then computed and two thresholding procedures applied to the amplitudes of these peak‐to‐peak differences (Goldberger & Ng, 2010).

The first procedure identified a threshold at each time *t* by computing the interquartile range of the combined set of smoothed voltages in the 5 s interval centred at *t* and a 5 s interval in the electrogram with no excitation. The threshold at *t* was set at 3 times this interquartile range. The second procedure identified thresholds over 100 ms intervals, using localized Otsu thresholding (Otsu, 1979), which uses the spread of values to identify an optimal threshold. The range of values used to calculate the Otsu threshold were all peak‐to‐peak differences above the initial threshold within the 0.5 s interval centred about the 100 ms interval. Application of the two thresholding procedures produced a sequence of times for each electrogram where the peak‐to‐peak difference at a given time is considered to represent an action potential spike. Bursts were identified from these spike times by setting a minimum burst length of 5 s, minimum frequency of spikes in the burst as 2 Hz, and maximum time difference between spikes in the burst as 1 s. An electrogram that contained a sequence of action potentials meeting the above criteria was considered to show bursting behaviour and was compared with other bursting electrograms to determine if they recorded the same excitation wave. Comparisons were performed between pairs of electrodes that were at most 1 cm apart. Electrograms were compared in pairs, with the assumption that if the excitation wave reaches both electrodes, then the sequence of spike times that appear in one electrogram will appear at a later point in time in the other electrogram. For each pair of electrograms the comparison was performed twice, one for each possible direction of travel. The comparison was performed by moving the spike times in one electrogram backwards in time and comparing 2 s time intervals of these displaced spike times to the spike times in the other electrogram. The range of physiologically plausible time displacements was taken to be from 50 to 1000 ms per cm distance between the electrodes, corresponding to a propagation speed between 1 and 20 cm s^−1^. These values were selected on the basis of previous observations (Rabotti & Mischi, 2015). A spike in this interval in either electrogram was deemed matched if it was within 20 ms of a spike in the other electrogram. An interval was deemed matched for a given time displacement if the proportion of spikes in the interval in both electrograms was greater than 0.7. The electrograms were deemed to match if the proportion of 2 s intervals that were matched for some time displacement was greater than 0.25. The matching algorithm was used to create distinct sets of electrodes recording the same excitation wave. For each set of electrodes in the recording, isochrone maps were generated for each set of electrodes in the recording, and colour‐coded to represent the time at which the excitation wave reached the electrodes.

### High‐resolution reconstruction of myometrial smooth muscle

Methods for serial sectioning of the tissue, image registration and identification of nuclei in the histological slides were described previously (Lutton *et al*. 2017). Briefly, the pinned tissue described above was fixed in formalin, embedded in paraffin and sliced into serial sections 5 μm thick. Slicing of the tissue was performed from the endometrial to the serosal side, causing most smooth muscle bundles in both circular and longitudinal layers of the myometrium to lie approximately parallel to the plane of slicing. Any slides that were too severely distorted by sectioning to register properly were discarded. These slides were stained with haematoxylin and eosin to identify the nuclei in the tissue. In each slide the nuclei of all cell types were automatically identified and the position, size and orientation were recorded (Lutton *et al*. 2017). In particular, the sizes and the orientation of the nuclei were recorded as the angle between the *x*‐axis and the major axis of the nucleus. Nuclei were first filtered by size, and any nuclei outside the range 10.4–62.1 μm^2^ were discarded. Nuclei were identified as being smooth muscle nuclei by imposing a rectangular area, centred at the nucleus of the given cell, of length 150 μm and width 30 μm with long axis aligned with the major axis of the nucleus. For a nucleus at point *p* with orientation angle *a*, the following average was computed:

$$H\left(p\right)=1/N\sum_{i\;=\;1}^{N}\left|\cos\left(a_{i}-a\right)\right|$$

where *a*_1_, … , *a_N_* are the orientation angles of all nuclei in the rectangle. If the orientation angle exceeded the threshold value 0.9, and the density of the rectangle was within the range 1000–3300 nuclei per mm^2^, then the nucleus at *p* was taken to be in a smooth muscle cell. Each slide was coarse‐grained to images with pixels of length 10 μm, with each pixel containing a nucleus identified as being in smooth muscle tissue assigned a value of 1. Any nuclei that were not identified as smooth muscle, but were contained in one or more rectangular areas of the above nuclei, were assigned the maximal value of |cos(*a_q_* − *a*)|, where *a_q_* is the orientation angle of the given nucleus and *a* is the orientation angle of a smooth muscle nucleus with associated rectangular area containing the given nucleus. These assigned values were placed in the pixels of the image containing the nuclei. The sparse image containing these assigned values was filled to represent smooth muscle cells by applying anisotropic Gaussian filters at each point of the image (Geusebroek *et al*. 2003). The anisotropic filters were rectangular in shape, with length 13 pixels and width 3 pixels (standard deviation 6 pixels along the long axis, 1 pixel along the short axis), and long axis along the local fibre direction, which was taken to be the direction of the nearest nucleus assigned a value in the previous step. Following assignment of pixel values in *x* and *y*, a smoothing step in the *z*‐axis was applied between slides, with a Gaussian kernel of standard deviation = 1 and length = 1.

MPPZs were identified manually by comparison of the computational reconstruction of the anatomy of electrically active areas with histological slides. For a given placental site, a cube in the reconstruction was selected as a seeding point and extending to approximately 1 cm away from the site with a lower threshold of 0.05 applied to the smoothed values to facilitate processing of the geometry of the tissue. Voxels were grouped into connected components, and all but the largest groups were discarded, as these represent areas not connected to the myometrium and therefore are electrically isolated. The decidua and mesometrial triangle were surrounded by myometrial smooth muscle in the histological sections.

Thus, within each slide plane, all areas interior to the thresholded pixels with area greater than 240 pixels (0.024 mm^2^) were taken to be part of the placentation site. Potential MPPZ structures were identified by dilating and subsequently eroding these interior points by 20 voxels (200 μm) (Toennies, 2012), so that as the points expand they engulf any structures projecting into the interior, with the structures then captured within edges for further processing. These structures were then segmented using hysteresis thresholding, of lower threshold 0.1 and upper threshold 0.25 (Nixon & Aguado, 2002). Structures were visualized by attribution of arbitrary colours in Mathematica 8.0 (Wolfram Research, Hanborough, UK) and selected by comparison to histological slides. Identified MPPZs were then re‐rendered in the full three‐dimensional volume.

## Results

In previous experiments on eight rats, with a total of 29 independent observations, we demonstrated that the majority of excitations recorded at the serosal surface originate from placental sites on the mesometrial border (Lammers *et al*. 2015). In these experiments, we were unable to determine the precise site of origin of the electrical activity. In order to correlate electrical activity with anatomical structure, we first repeated these recordings of electrical potentials from the serosal surface of rat myometrium.

**Figure 2:**
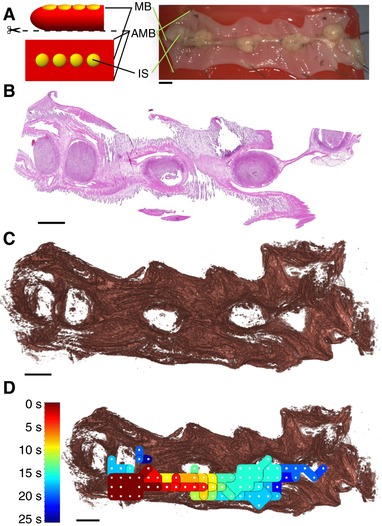
Experimental design *A*, three rat uteri were cut along the anti‐mesometrial border (AMB) and pinned with the serosal side facing upwards, positioning the mesometrial border (MB), along with the implantation sites (IS), in the centre of the tissue. *B*, each uterus was fixed in formalin, embedded in paraffin, sectioned into 5 μm serial sections, and stained with haematoxylin and eosin. *C*, detailed *in silico* reconstructions of the uteri were generated from these serial sections using a semi‐automated image analysis pipeline. *D*, multi‐electrode array recordings were performed prior to fixation, which were processed to generate isochrone maps that represent the spread of electrical activity. These isochrone maps were compared to the reconstructed tissue to identify structural features in the tissue that affect the initiation and termination of the excitation waves. Colours in the isochrone map correspond to the time at which the excitation wave reaches the given area in the tissue (colour key on the left). All images representing tissue are oriented with the ovarian end of the tissue on the left. Scale bars represent 5 mm.

After recording three 30 min time series of spontaneous activity per preparation, each taken from a different animal, the tissues were processed to capture the 3‐dimensional histological microarchitecture as described previously (Lutton *et al*. 2017). Histological structure and electrical activity were subsequently collated to identify the anatomical sites where electrical excitation originates (Fig. 2).

### Uterine structure

The general higher order structure of all samples analysed was consistent with that of a highly ordered inner circular layer of myometrial smooth muscle, which surrounds the decidualized stromal cells lining the lumen. The outer, subserosal, layer of the myometrial smooth muscle has a longitudinal orientation and is separated from the inner circular layer by connective tissue and vasculature. In rodents, this general structure is well described, and is patterned during early postnatal development (Brody & Cunha, 1989).

**Figure 3:**
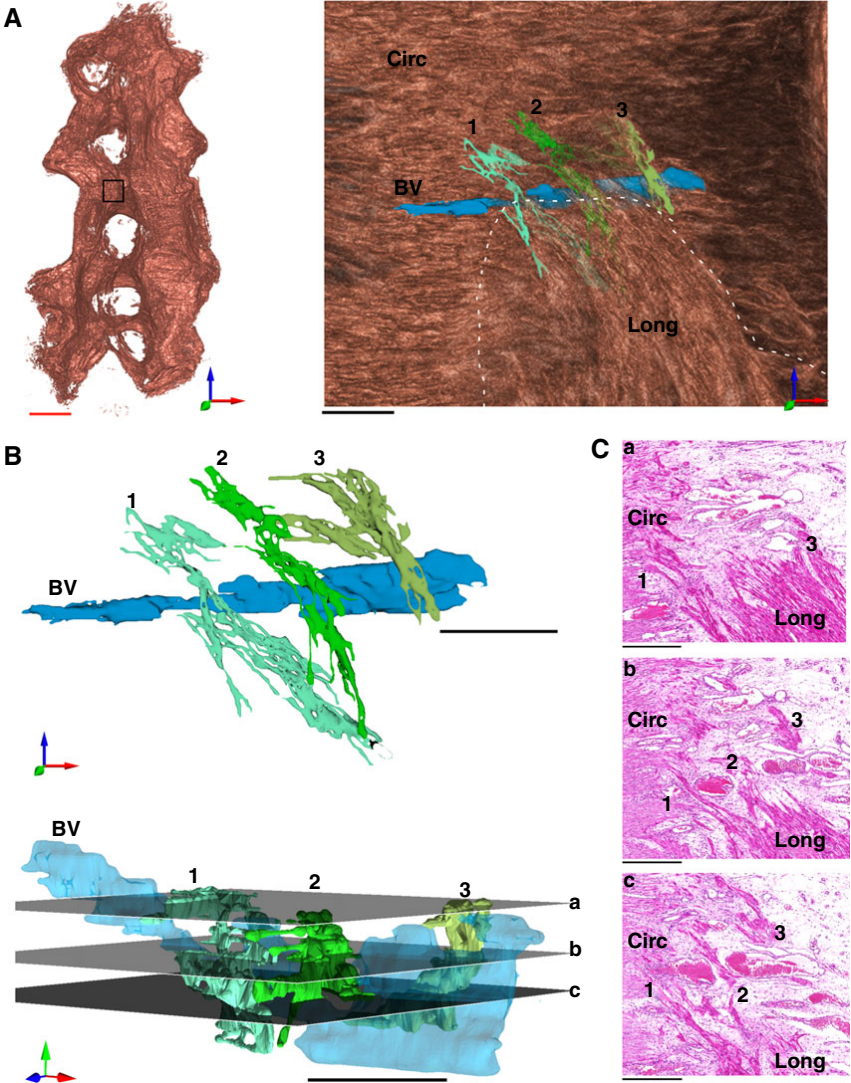
Myometrial smooth muscle bridges connect the longitudinal and circular layers *A*, 3‐dimensional reconstruction of the bridge‐like structures within the myometrium. Inset box in close proximity to the mesometrial border observed from a lateral viewpoint shows bridges (1, 2, 3) surrounding a blood vessel (BV) and joining the circular (Circ) and longitudinal (Long) myometrium. The dotted line represents a graphical cut‐away boundary removing the longitudinal layer to reveal the underlying circular layer. Bridge 1, cyan; bridge 2, dark green; bridge 3, light green. *B*, bridges depicted in *A* isolated for visualization. Planes *a*, *b*, and *c* can be observed in the histological sections (haematoxylin and eosin) depicted in *C*. *C*, histological sections of planes *a*, *b* and *c*. Bridges 1, 2 and 3 can be observed in cross section, within the vasculature of the intermediate layer. Black scale bars represent 500 μm; red scale bar represents 5 mm.

**Figure 4:**
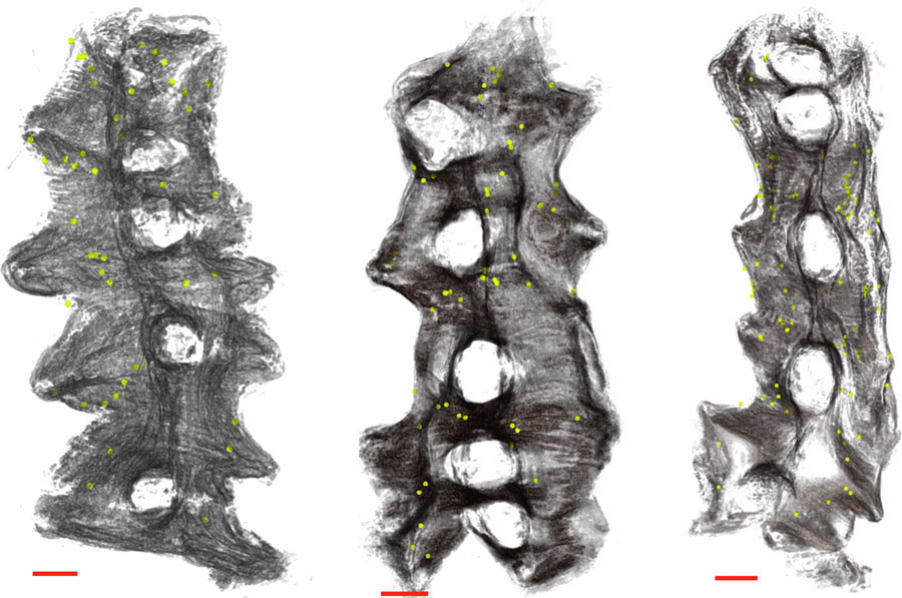
Reconstruction of bridge positions (green dots) in the uterine preparations Bridges are numerous and distributed throughout the tissue. Scale bar represents 5 mm.

*In silico* tissue reconstruction visualized and charted the three‐dimensional fibrous structure at high resolution (Lutton *et al*. 2017), allowing us to discover novel anatomical features of the myometrium of functional significance. Detailed analysis revealed bridge‐like structures of myometrial smooth muscle that were invariably associated with the vascular bed of the intermediate layer and which form direct connections between the longitudinal and circular layers of the myometrium (Fig. 3). The observed bridges were numerous and scattered throughout the tissue (Fig. 4).

### Electrode array recordings and identification of myometrial placental pacemaker zones

**Figure 5:**
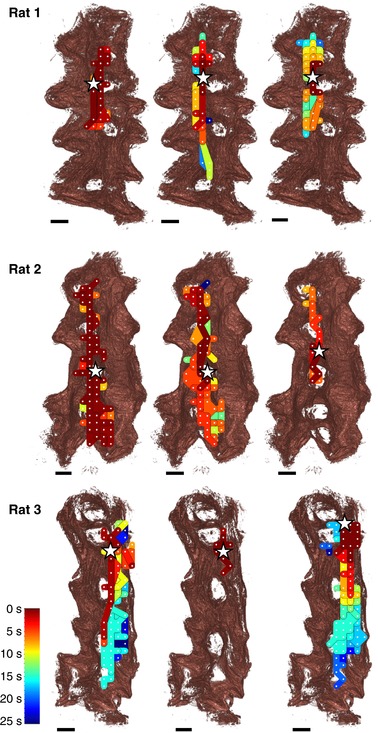
Isochrone maps generated from the multi‐electrode array recordings in each of the uteri, superimposed onto the *in silico* reconstructions of the tissue Each row shows isochrone maps of recordings taken from each of the rat uteri, where each recording covers 1 min of activity, and consecutive recordings are separated by 10 min. Isochrones are coloured based on the time that the excitation wave reached the given area according to the key shown, with each isochrone representing a 200 ms time band. The electrode closest to where the excitation wave was initiated in each recording was defined by the earliest time point of the start of a burst within a matched network of propagation and is indicated by a white star. Initiation points all lie along the mesometrial border in close proximity to the implantation sites. Excitation waves propagate along the mesometrial border in all but one recording (exception shown bottom right). All recordings show excitation confined to one side of or along the mesometrial border, with many exhibiting excitation spreading across the implantation sites. Uteri are shown here with the ovarian end at the top of the image. Scale bars represent 5 mm.

We analysed the spatio‐temporal electrode array data in combination with the anatomical data to determine where electrical activity was originating. The electrode‐array data were processed using an algorithm, which identified similarities in the waveform of a train of action potentials that were used to cross‐correlate signals at different locations in the array, allowing a given activation event to be tracked in time and space. In total 382 bursts were analysed with mean burst duration of 39.2 ±14.6 s (mean ± SD). Figure 4 presents the results as isochrone maps, superimposed on *in silico* reconstructions of the uteri. For each activation event, the electrode in closest proximity to the putative initiation point was identified and the local anatomical area was examined for common features that were confirmed for direct connectivity. In eight of the nine recordings, excitation was initiated in close proximity to a placental site, with subsequent activity spreading along the mesometrial border. The final recording in the third tissue sample (bottom right in Fig. 5) exhibited a different pattern of excitation, with activity initiated at the ovarian end of the tissue and spreading away from the mesometrial border; such excitations, though rarer than placental bed events, have been observed previously in these preparations (Lammers *et al*. 2015).

**Figure 6:**
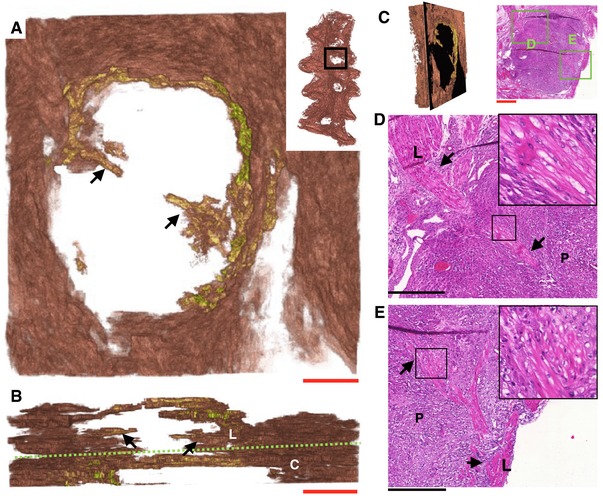
Electrical activity originates in smooth muscle fibres at the myometrial‐placental interface *A*, three‐dimensional reconstruction of myometrial bundles (arrows) present in the placental bed, observed from the serosal side of the tissue. *B*, the same bundles (arrows) viewed from the cervical end of the tissue, with the approximate boundaries between the longitudinal (L) and circular (C) layers indicated by a green dashed line, showing that the bundles are attached to the longitudinal myometrium. *C*, comparison of reconstructed tissue (*A* and *B*) and histological slides (*D* and *E*). *D* and *E*, histology of bundles shown in *A* and *B*, with the points of attachment to the longitudinal myometrium and farthest extension into the placenta indicated by arrows. Insets show more detail of the boxes indicated, in each case revealing that the cells in these structures have a stellate morphology. The initiation points for electrical activity in this uterus occur consistently in close proximity to the structure shown in *D*. All images (excluding *B*) are oriented with the ovarian end of the tissue at the top of the image. C, circular myometrium; L, longitudinal myometrium; P, placenta. Red scale bars represent 1 mm; black scale bars represent 500 μm.

**Figure 7:**
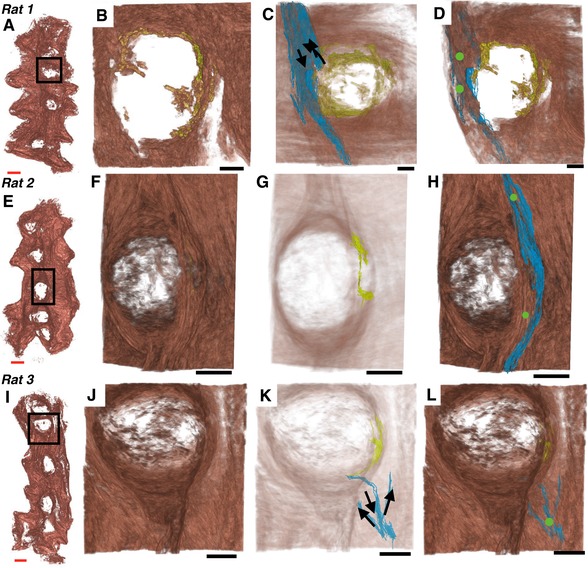
MPPZs associated with excitation activity in relation to the surrounding reconstructed tissue *A*, *E* and *I*, location of the placenta containing the MPPZ. *B*, *F* and *J*, high‐resolution reconstruction of the myometrium adjacent to the placenta containing the MPPZ. The MPPZ is shown in *B*, having been identified in the reconstruction algorithm (see also Fig. 5). *C*, *G* and *K*, MPPZ (yellow) with reconstructed tissue shown at reduced opacity for clarity. The proposed path of excitation (blue) is shown in *C* and *K* with the proposed direction of excitation (arrows). The proposed path is omitted from *G* in order to show the MPPZ clearly. *D*, *H* and *L*, MPPZ (yellow), proposed path of excitation (blue) and the location of electrodes identified as initiation points in the recordings (green circles). Longitudinal myometrium was present superficial to the MPPZs; it was therefore necessary to identify the path that an excitation wave follows from the MPPZ to the serosal surface, where the electrodes were situated. The proposed paths of excitation were obtained by visual inspection of the histological slides, using the 3‐dimensional reconstructions to identify corresponding bundles between slides. The proposed path in the third sample (*K* and *L*) is restricted to the bundle connecting the MPPZ to the myometrium and the shortest unbroken path from this bundle to two adjacent electrodes; adjacent bundles are connected to these bundles, but have been omitted for clarity. Red scale bar represents 5 mm; black scale bars represent 2 mm.

Investigation of the histological slides at these initiation points revealed myometrial bundles in the placenta that were contiguous with the longitudinal layer (Fig. 6). High‐resolution three‐dimensional reconstructions of these structures revealed finger‐like projections of myometrium in close proximity to all placental beds (*n *= 14). When visualized on the haematoxylin and eosin histology sections, myometrial smooth muscle cells within these regions were distinguished by their stellate morphology from cells within the same bundle but situated outside of the placenta (Fig. 5 *D* and *E*). These bundles appear to be modified bridge‐like structures that have been captured by the invading placenta. Combination of the electrical recordings with the anatomical structure of the MPPZs and surrounding myometrial smooth muscle cell network allowed reconstruction of each event, demonstrating a distinct path of excitation from the MPPZ to the longitudinal fibres (Fig. 7).

## Discussion

Parturition in the rodent is initiated by a combination of endocrine and paracrine signals. Central to the onset of labour is the systemic withdrawal of progesterone caused by prostanoid‐induced collapse of the corpus luteum (Bonventre *et al*. 1997; Sugimoto *et al*. 1997; Gross *et al*. 1998). The fall in circulating progesterone levels, combined with increased local progesterone resistance (Condon *et al*. 2003; Renthal *et al*. 2010), activates genes that encode contraction‐associated proteins, such as connexin 43 (Garfield *et al*. 1977) and the oxytocin receptor (Fuchs *et al*. 1982; Fuchs *et al*. 1983), which both render the myometrium responsive to excitation stimuli and synchronize contractions. Concomitant with alterations in susceptibility to stimulation, decidual senescence (Hirota *et al*. 2011; Cha *et al*. 2013) and fetal signals (Condon *et al*. 2004) increase prostanoid synthesis promoting electrical excitation, voltage‐gated calcium entry and contraction (Parkington *et al*. 1999*b*), but the precise site of activation has, until now, remained unknown.

In this study, we combined multi‐electrode recordings with high‐resolution anatomical reconstruction to demonstrate that electrical potentials predominantly originate at specialized interfaces of the myometrium and placenta. Moreover, our results suggest that these MPPZs form a conduit between each implanted feto‐placental unit and the broader myometrial smooth muscle network. The spatial organization of the bundles of myometrial smooth muscle in the area of vascular and connective tissue between the longitudinal and circular layers appears to be of particular significance. Bridge‐like structures that apparently occur randomly across the tissue, but always in close proximity to the vasculature, form connections between the inner circular myometrium and the outer longitudinal myometrium.

The mesometrial–antimesometrial axis governs orientation in rodents; implantation into the antimesometrial lumen on day 4 after formation of the vaginal plug orientates the blastocyst for placentation into the mesometrial border (Daikoku *et al*. 2011). The asymmetry of implantation is encoded by a Wnt5a gradient across the uterine lumen that creates a timed evagination and subsequent implantation crypt (Cha *et al*. 2014). The regular spacing of the implantation crypts is directed by planar cell polarity signalling, as mice deficient in the non‐canonical Wnt intermediary Vangl2 exhibit defective crypt formation and severely compromised pregnancy outcomes (Yuan *et al*. 2016). Our data suggests that the asymmetry of patterning, combined with the association of bridges with vasculature, promotes co‐localization of implantation with the vascular supply of the mesometrial axis, and the bridge‐like structures of myometrium that provide electrical access to the entire myometrial network. It remains unclear whether the bridge‐like structures are formed postnatally concurrently with the circular and longitudinal layers (Brody & Cunha, 1989) or at the same time as the crypt structures of the lumen (Daikoku *et al*. 2011; Cha *et al*. 2014; Yuan *et al*. 2016). However, as the vascular network forms before the circular and longitudinal layers (Brody & Cunha, 1989), we surmise that bridges form postnatally through the paths created by the vascular bed.

The myometrial fibres we observe as the MPPZs are analogous to those described by Pijnenborg and coworkers in an immuno‐histochemical study of placental sites in the late‐pregnant rat (Vercruysse *et al*. 2006). Using cytokeratin as a marker for invading interstitial trophoblast, and α‐actin as a marker for myometrial smooth muscle, the study identified invasion of the lateral longitudinal myometrium by trophoblast as a ubiquitous event by day 20 and 21 of pregnancy. The invading interstitial trophoblast front originates from glycogen cell islands at the trophospongial–decidual junction, near the maternal channel, and temporally coincides with lower interstitial trophoblast number in the decidua and progesterone withdrawal. It is tempting to speculate that this event is timed to coincide with the onset of parturition to establish MPPZs capable of initiating uterine contractions in response to decidual senescence.

Our computational reconstructions of the MPPZ regions suggest that they originate from myometrial bundles that have become rearranged during extra‐villous trophoblast invasion. This implies that poor implantation, or implantation outside of the mesometrial axis (i.e. not associated with the vascular bed), would lead to poor pregnancy outcomes, as has been demonstrated experimentally in several genetic mouse models (Song *et al*. 2002; Ye *et al*. 2005; Sun *et al*. 2012).

The anatomical restructuring of the myometrium and the formation of the MPPZs during pregnancy described in this study precedes the gestation‐dependent alteration in electrical excitability that is conserved across all mammals tested to date (Casteels & Kuriyama, 1965; Parkington *et al*. 1999*a*). These changes are mediated by the expression of different classes of potassium channel (Brainard *et al*. 2007), some of which control resting membrane potential (McCloskey *et al*. 2014) whilst others modulate the action potential waveform to allow for more forceful contractions of longer duration (Khan *et al*. 1993; Knock *et al*. 1999; Pierce *et al*. 2008; Greenwood *et al*. 2009; Parkington *et al*. 2014; Atia *et al*. 2016). Thus, the timely activation of the MPPZ at each feto‐maternal interface, in a coupled and excitable electrical network, provides an elegant solution for driving contraction from the placental site towards the cervix to facilitate delivery of multiple fetuses in a polytocus uterus. It is currently not known whether these MPPZ regions exist in the placentas of a singleton uterus such as the human. We have recently demonstrated that there are substantial myometrial fibres that originate from the larger outer body of the human uterus (the stratum supra vasculare), traverse the stratum vasculare and terminate in the inner junctional zone at a depth consistent with placental invasion (Lutton *et al*. 2017). Such fibres could form a conduit between the myometrial placental interface and the larger, main body of the uterus. In conclusion, we have shown that electrical activity in the rat myometrium originates from MPPZs and that the spatial organization of these areas likely promotes coordinated delivery of fetuses in a polytocus uterus.

## Additional information

### Competing interests

The authors declare no conflicts of interest.

### Author contributions

E.J.L. undertook some histology, wrote all computer algorithms, prepared figures and co‐wrote the manuscript. W.J.E.P.L. co‐conceived the project and undertook the electrode array recordings. S.J. undertook the histology work. H.A.B. co‐conceived the project, co‐supervized the generation of the computer algorithms and co‐wrote the manuscript. A.M.B. co‐conceived the project, co‐supervized the generation of the computer algorithms, co‐prepared figures, co‐wrote the manuscript and obtained funding. All authors input into the data analysis and the manuscript. All authors have read and approved the final version of this manuscript and agree to be accountable for all aspects of the work in ensuring that questions related to the accuracy or integrity of any part of the work are appropriately investigated and resolved. All persons designated as authors qualify for authorship, and all those who qualify for authorship are listed.

### Funding

E.J.L., A.M.B. and H.A.B. were supported by a grant from the Medical Research Council (UK), grant number G0900208‐2. The funders had no role in study design, data collection and analysis, decision to publish, or preparation of the manuscript.

## References

[tjp12997-bib-0001] Atia J , McCloskey C , Shmygol AS , Rand DA , van den Berg HA & Blanks AM (2016). Reconstruction of cell surface densities of ion pumps, exchangers, and channels from mRNA expression, conductance kinetics, whole‐cell calcium, and current‐clamp voltage recordings, with an application to human uterine smooth muscle cells. PLoS Comput Biol 12, e1004828. 27105427 10.1371/journal.pcbi.1004828 PMC4841602

[tjp12997-bib-0002] Bonventre JV , Huang Z , Taheri MR , O'Leary E , Li E , Moskowitz MA & Sapirstein A (1997). Reduced fertility and postischaemic brain injury in mice deficient in cytosolic phospholipase A2. Nature 390, 622–625. 9403693 10.1038/37635

[tjp12997-bib-0003] Brainard AM , Korovkina VP & England SK (2007). Potassium channels and uterine function. Semin Cell Dev Biol 18, 332–339. 17596977 10.1016/j.semcdb.2007.05.008 PMC2012947

[tjp12997-bib-0004] Brody JR & Cunha GR (1989). Histologic, morphometric, and immunocytochemical analysis of myometrial development in rats and mice: I. Normal development. Am J Anat 186, 1–20. 2782286 10.1002/aja.1001860102

[tjp12997-bib-0005] Burden RL & Faires JD (2005). Numerical Analysis. Cengage Learning, Boston, MA, USA.

[tjp12997-bib-0006] Casteels R & Kuriyama H (1965). Membrane potential and ionic content in pregnant and non‐pregnant rat myometrium. J Physiol 177, 263–287. 14302154 10.1113/jphysiol.1965.sp007591 PMC1357244

[tjp12997-bib-0007] Cha J , Bartos A , Egashira M , Haraguchi H , Saito‐Fujita T , Leishman E , Bradshaw H , Dey SK & Hirota Y (2013). Combinatory approaches prevent preterm birth profoundly exacerbated by gene‐environment interactions. J Clin Invest 123, 4063–4075. 23979163 10.1172/JCI70098 PMC3754274

[tjp12997-bib-0008] Cha J , Bartos A , Park C , Sun X , Li Y , Cha SW , Ajima R , Ho HY , Yamaguchi TP & Dey SK (2014). Appropriate crypt formation in the uterus for embryo homing and implantation requires Wnt5a‐ROR signaling. Cell Rep 8, 382–392. 25043182 10.1016/j.celrep.2014.06.027 PMC4120233

[tjp12997-bib-0009] Condon JC , Jeyasuria P , Faust JM & Mendelson CR (2004). Surfactant protein secreted by the maturing mouse fetal lung acts as a hormone that signals the initiation of parturition. Proc Natl Acad Sci U S A 101, 4978–4983. 15044702 10.1073/pnas.0401124101 PMC387359

[tjp12997-bib-0010] Condon JC , Jeyasuria P , Faust JM , Wilson JW & Mendelson CR (2003). A decline in the levels of progesterone receptor coactivators in the pregnant uterus at term may antagonize progesterone receptor function and contribute to the initiation of parturition. Proc Natl Acad Sci U S A 100, 9518–9523. 12886011 10.1073/pnas.1633616100 PMC170950

[tjp12997-bib-0011] Daikoku T , Cha J , Sun X , Tranguch S , Xie H , Fujita T , Hirota Y , Lydon J , DeMayo F , Maxson R & Dey SK (2011). Conditional deletion of Msx homeobox genes in the uterus inhibits blastocyst implantation by altering uterine receptivity. Dev Cell 21, 1014–1025. 22100262 10.1016/j.devcel.2011.09.010 PMC3241866

[tjp12997-bib-0012] Fuchs AR , Fuchs F , Husslein P , Soloff MS & Fernstrom MJ (1982). Oxytocin receptors and human parturition: a dual role for oxytocin in the initiation of labor. Science 215, 1396–1398. 6278592 10.1126/science.6278592

[tjp12997-bib-0013] Fuchs AR , Periyasamy S , Alexandrova M & Soloff MS (1983). Correlation between oxytocin receptor concentration and responsiveness to oxytocin in pregnant rat myometrium: effects of ovarian steroids. Endocrinology 113, 742–749. 6872947 10.1210/endo-113-2-742

[tjp12997-bib-0014] Garfield RE , Blennerhassett MG & Miller SM (1988). Control of myometrial contractility: role and regulation of gap junctions. Oxf Rev Reprod Biol 10, 436–490. 3072507

[tjp12997-bib-0015] Garfield RE & Maner WL (2007). Physiology and electrical activity of uterine contractions. Semin Cell Dev Biol 18, 289–295. 17659954 10.1016/j.semcdb.2007.05.004 PMC2048588

[tjp12997-bib-0016] Garfield RE , Sims S & Daniel EE (1977). Gap junctions: their presence and necessity in myometrium during parturition. Science 198, 958–960. 929182 10.1126/science.929182

[tjp12997-bib-0017] Geusebroek JM , Smeulders AWM & Van De Weijer J ( 2003). Fast anisotropic gauss filtering. IEEE Trans Image Process 12, 938–943. 18237967 10.1109/TIP.2003.812429

[tjp12997-bib-0018] Goldberger JJ & Ng J (2010). Practical Signal and Image Processing in Clinical Cardiology. Springer, London.

[tjp12997-bib-0019] Greenwood IA , Yeung SY , Tribe RM & Ohya S (2009). Loss of functional K^+^ channels encoded by *ether‐à‐go‐go‐related* genes in mouse myometrium prior to labour onset. J Physiol 587, 2313–2326. 19332483 10.1113/jphysiol.2009.171272 PMC2697300

[tjp12997-bib-0020] Gross GA , Imamura T , Luedke C , Vogt SK , Olson LM , Nelson DM , Sadovsky Y & Muglia LJ (1998). Opposing actions of prostaglandins and oxytocin determine the onset of murine labor. Proc Natl Acad Sci U S A 95, 11875–11879. 9751758 10.1073/pnas.95.20.11875 PMC21733

[tjp12997-bib-0021] Hammad FT , Stephen B , Lubbad L , Morrison JF & Lammers WJ (2014). Macroscopic electrical propagation in the guinea pig urinary bladder. Am J Physiol Renal Physiol 307, F172–F182. 24899061 10.1152/ajprenal.00215.2014

[tjp12997-bib-0022] Hirota Y , Cha J , Yoshie M , Daikoku T & Dey SK (2011). Heightened uterine mammalian target of rapamycin complex 1 (mTORC1) signaling provokes preterm birth in mice. Proc Natl Acad Sci U S A 108, 18073–18078. 22025690 10.1073/pnas.1108180108 PMC3207648

[tjp12997-bib-0023] Khan RN , Smith SK , Morrison JJ & Ashford ML (1993). Properties of large‐conductance K^+^ channels in human myometrium during pregnancy and labour. Proc Biol Sci 251, 9–15. 8094568 10.1098/rspb.1993.0002

[tjp12997-bib-0024] Knock GA , Smirnov SV & Aaronson PI (1999). Voltage‐gated K^+^ currents in freshly isolated myocytes of the pregnant human myometrium. J Physiol 518, 769–781. 10420013 10.1111/j.1469-7793.1999.0769p.x PMC2269461

[tjp12997-bib-0025] Lammers WJ & Stephen B (2008). Origin and propagation of individual slow waves along the intact feline small intestine. Exp Physiol 93, 334–346. 18156170 10.1113/expphysiol.2007.039180

[tjp12997-bib-0026] Lammers WJ , Stephen B , Al‐Sultan MA , Subramanya SB & Blanks AM (2015). The location of pacemakers in the uteri of pregnant guinea pigs and rats. Am J Physiol Regul Integr Comp Physiol 309, R1439–R1446. 26377559 10.1152/ajpregu.00187.2015

[tjp12997-bib-0027] Lammers WJ , Ver Donck L , Stephen B , Smets D & Schuurkes JA (2009). Origin and propagation of the slow wave in the canine stomach: the outlines of a gastric conduction system. Am J Physiol Gastrointest Liver Physiol 296, G1200–G1210. 19359425 10.1152/ajpgi.90581.2008

[tjp12997-bib-0028] Lutton EJ , Lammers WJ , James S , van den Berg HA & Blanks AM (2017). A computational method for three‐dimensional reconstruction of the microarchitecture of myometrial smooth muscle from histological sections. PLoS One 12, e0173404. 28301486 10.1371/journal.pone.0173404 PMC5354307

[tjp12997-bib-0029] McCloskey C , Rada C , Bailey E , McCavera S , van den Berg HA , Atia J , Rand DA , Shmygol A , Chan YW , Quenby S , Brosens JJ , Vatish M , Zhang J , Denton JS , Taggart MJ , Kettleborough C , Tickle D , Jerman J , Wright P , Dale T , Kanumilli S , Trezise DJ , Thornton S , Brown P , Catalano R , Lin N , England SK & Blanks AM (2014). The inwardly rectifying K^+^ channel KIR7.1 controls uterine excitability throughout pregnancy. EMBO Mol Med 6, 1161–1174. 25056913 10.15252/emmm.201403944 PMC4197863

[tjp12997-bib-0030] Nixon M & Aguado A (2002). Feature Extraction & Image Processing. Newnes, Oxford.

[tjp12997-bib-0031] Otsu N ( 1979). A threshold selection method from gray‐level histograms. IEEE Trans Syst Man Cybern 9, 62–66.

[tjp12997-bib-0032] Parkington HC , Stevenson J , Tonta MA , Paul J , Butler T , Maiti K , Chan EC , Sheehan PM , Brennecke SP , Coleman HA & Smith R (2014). Diminished hERG K^+^ channel activity facilitates strong human labour contractions but is dysregulated in obese women. Nat Commun 5, 4108. 24937480 10.1038/ncomms5108

[tjp12997-bib-0033] Parkington HC , Tonta MA , Brennecke SP & Coleman HA (1999a). Contractile activity, membrane potential, and cytoplasmic calcium in human uterine smooth muscle in the third trimester of pregnancy and during labor. Am J Obstet Gynecol 181, 1445–1451. 10601927 10.1016/s0002-9378(99)70390-x

[tjp12997-bib-0034] Parkington HC , Tonta MA , Davies NK , Brennecke SP & Coleman HA (1999b). Hyperpolarization and slowing of the rate of contraction in human uterus in pregnancy by prostaglandins E2 and F2α: involvement of the Na^+^ pump. J Physiol 514, 229–243. 9831729 10.1111/j.1469-7793.1999.229af.x PMC2269046

[tjp12997-bib-0035] Pierce SL , Kresowik JDK , Lamping KG & England SK (2008). Overexpression of SK3 channels dampens uterine contractility to prevent preterm labor in mice. Biol Reprod 78, 1058–1063. 18305226 10.1095/biolreprod.107.066423 PMC2930016

[tjp12997-bib-0036] Rabotti C & Mischi M (2015). Propagation of electrical activity in uterine muscle during pregnancy: a review. Acta Physiol (Oxf) 213, 406–416. 25393600 10.1111/apha.12424

[tjp12997-bib-0037] Renthal NE , Chen CC , Williams KC , Gerard RD , Prange‐Kiel J & Mendelson CR (2010). miR‐200 family and targets, ZEB1 and ZEB2, modulate uterine quiescence and contractility during pregnancy and labor. Proc Natl Acad Sci U S A 107, 20828–20833. 21079000 10.1073/pnas.1008301107 PMC2996411

[tjp12997-bib-0038] Song H , Lim H , Paria BC , Matsumoto H , Swift LL , Morrow J , Bonventre JV & Dey SK (2002). Cytosolic phospholipase A2alpha is crucial [correction of A2alpha deficiency is crucial] for ‘on‐time’ embryo implantation that directs subsequent development. Development 129, 2879–2889. 12050136 10.1242/dev.129.12.2879

[tjp12997-bib-0039] Sugimoto Y , Yamasaki A , Segi E , Tsuboi K , Aze Y , Nishimura T , Oida H , Yoshida N , Tanaka T , Katsuyama M , Hasumoto K , Murata T , Hirata M , Ushikubi F , Negishi M , Ichikawa A & Narumiya S ( 1997). Failure of parturition in mice lacking the prostaglandin F receptor. Science 277, 681–683. 9235889 10.1126/science.277.5326.681

[tjp12997-bib-0040] Sun X , Zhang L , Xie H , Wan H , Magella B , Whitsett JA & Dey SK (2012). Kruppel‐like factor 5 (KLF5) is critical for conferring uterine receptivity to implantation. Proc Natl Acad Sci U S A 109, 1145–1150. 22233806 10.1073/pnas.1118411109 PMC3268277

[tjp12997-bib-0041] Toennies KD ( 2012). Guide to Medical Image Analysis. Springer, Berlin, Heidelberg.

[tjp12997-bib-0042] Vercruysse L , Caluwaerts S , Luyten C & Pijnenborg R (2006). Interstitial trophoblast invasion in the decidua and mesometrial triangle during the last third of pregnancy in the rat. Placenta 27, 22–33. 16310034 10.1016/j.placenta.2004.11.004

[tjp12997-bib-0043] Ye X , Hama K , Contos JJ , Anliker B , Inoue A , Skinner MK , Suzuki H , Amano T , Kennedy G , Arai H , Aoki J & Chun J (2005). LPA3‐mediated lysophosphatidic acid signalling in embryo implantation and spacing. Nature 435, 104–108. 15875025 10.1038/nature03505 PMC1369590

[tjp12997-bib-0044] Yuan J , Cha J , Deng W , Bartos A , Sun X , Ho HH , Borg JP , Yamaguchi TP , Yang Y & Dey SK (2016). Planar cell polarity signaling in the uterus directs appropriate positioning of the crypt for embryo implantation. Proc Natl Acad Sci U S A 113, E8079–E8088. 27911818 10.1073/pnas.1614946113 PMC5167210

